# HTLV-1 persistence in vivo: clonality, dynamics and immune response

**DOI:** 10.1186/1742-4690-8-S1-A97

**Published:** 2011-06-06

**Authors:** Charles R M Bangham, Nicolas Gillet, Anat Melamed, Frédéric Toulza, Aileen Rowan, Daniel Laydon, Graham P Taylor, Becca Asquith

**Affiliations:** 1 Department of Immunology, Imperial College, London W2 1PG, UK; 2 Department of Genitourinary Medicine and Communicable Diseases, Wright-Fleming Institute, Imperial College London, London, W2 1PG, UK

## 

HTLV-1 propagates in the host by two parallel mechanisms: infectious spread, i.e. direct transfer of virions from cell to cell via the virological synapse; and mitotic spread, i.e. proliferation of HTLV-1-infected T cell clones. During chronic infection, mitotic spread predominates, generating very large clones in some individuals. Yet the reasons for the remarkable variation within and between hosts in the abundance of HTLV-1-infected clones remain unknown.

We aim to identify and quantify the selection forces that determine the size of HTLV-1-infected T cell clones in vivo and hence the risk of the associated inflammatory and malignant diseases. We shall summarize evidence from host and viral genetics, cellular immunology and high-throughput proviral integration site analysis that:

1. The ‘quality’ or efficiency of a person’s genetically-determined cytotoxic T lymphocyte (CTL) response to the HBZ protein is a major determinant of that person’s proviral load and the risk of inflammatory diseases.

2. The chief determinant of CTL quality is the host genotype in HLA Class 1 and Killer cell Immunoglobulin-like Receptor (KIR) loci.

3. The CTL response to the Tax protein is immunodominant but does not protect against HAM/TSP.

4. The site and orientation of the HTLV-1 provirus integrated in the T cell genome determine the size of each T cell clone in the host.

5. Negative selection on the virus dominates during chronic infection: we postulate that this selection is exerted by CTLs.

